# Enantiomer‐specific pharmacokinetics of D,L‐3‐hydroxybutyrate: Implications for the treatment of multiple acyl‐CoA dehydrogenase deficiency

**DOI:** 10.1002/jimd.12365

**Published:** 2021-02-15

**Authors:** Willemijn J. van Rijt, Johan L. K. Van Hove, Frédéric M. Vaz, Rick Havinga, Derk P. Allersma, Tanja R. Zijp, Jirair K. Bedoyan, M. R. Heiner‐Fokkema, Dirk‐Jan Reijngoud, Michael T. Geraghty, Ronald J. A. Wanders, Maaike H. Oosterveer, Terry G. J. Derks

**Affiliations:** ^1^ University of Groningen, University Medical Center Groningen, Beatrix Children's Hospital, Section of Metabolic Diseases Groningen The Netherlands; ^2^ Section of Clinical Genetics and Metabolism, Department of Pediatrics University of Colorado, Children's Hospital Colorado Aurora Colorado USA; ^3^ Departments of Clinical Chemistry and Pediatrics, Amsterdam Gastroenterology Endocrinology Metabolism Laboratory Genetic Metabolic Diseases, Amsterdam UMC, University of Amsterdam Amsterdam The Netherlands; ^4^ Core Facility Metabolomics, Amsterdam UMC Amsterdam The Netherlands; ^5^ Department of Pediatrics Groningen University of Groningen, University Medical Center Groningen Groningen The Netherlands; ^6^ Department of Clinical Pharmacy and Pharmacology University of Groningen, University Medical Center Groningen Groningen The Netherlands; ^7^ Department of Genetics and Genome Sciences, Case Western Reserve University and Center for Inherited Disorders of Energy Metabolism University Hospitals, Cleveland Medical Center Cleveland Ohio USA; ^8^ Laboratory of Metabolic Diseases, Department of Laboratory Medicine University of Groningen, University Medical Center Groningen Groningen The Netherlands; ^9^ Division of Metabolics and Newborn Screening, Department of Pediatrics Children's Hospital of Eastern Ontario Ottawa Canada

**Keywords:** 3‐hydroxybutyrate, enantiomer, inborn error of metabolism, ketone bodies, multiple acyl‐CoA dehydrogenase deficiency, pharmacokinetics

## Abstract

D,L‐3‐hydroxybutyrate (D,L‐3‐HB, a ketone body) treatment has been described in several inborn errors of metabolism, including multiple acyl‐CoA dehydrogenase deficiency (MADD; glutaric aciduria type II). We aimed to improve the understanding of enantiomer‐specific pharmacokinetics of D,L‐3‐HB. Using UPLC‐MS/MS, we analyzed D‐3‐HB and L‐3‐HB concentrations in blood samples from three MADD patients, and blood and tissue samples from healthy rats, upon D,L‐3‐HB salt administration (patients: 736‐1123 mg/kg/day; rats: 1579‐6317 mg/kg/day of salt‐free D,L‐3‐HB). D,L‐3‐HB administration caused substantially higher L‐3‐HB concentrations than D‐3‐HB. In MADD patients, both enantiomers peaked at 30 to 60 minutes, and approached baseline after 3 hours. In rats, D,L‐3‐HB administration significantly increased *C*_max_ and AUC of D‐3‐HB in a dose‐dependent manner (controls vs ascending dose groups for *C*_max_: 0.10 vs 0.30‐0.35‐0.50 mmol/L, and AUC: 14 vs 58‐71‐106 minutes*mmol/L), whereas for L‐3‐HB the increases were significant compared to controls, but not dose proportional (*C*_max_: 0.01 vs 1.88‐1.92‐1.98 mmol/L, and AUC: 1 vs 380‐454‐479 minutes*mmol/L). L‐3‐HB concentrations increased extensively in brain, heart, liver, and muscle, whereas the most profound rise in D‐3‐HB was observed in heart and liver. Our study provides important knowledge on the absorption and distribution upon oral D,L‐3‐HB. The enantiomer‐specific pharmacokinetics implies differential metabolic fates of D‐3‐HB and L‐3‐HB.

**SYNOPSIS**

The enantiomer‐specific pharmacokinetics upon a single, oral dose of racemic D,L‐3‐HB implies differential metabolic fates of D‐3‐HB and L‐3‐HB.

## 1 INTRODUCTION

Upon prolonged fasting, increased mitochondrial fatty acid oxidation (FAO) fuels energy production in many tissues and hepatic ketogenesis in healthy individuals. When glucose availability is limited, ketone bodies (KB) acetoacetate (AcAc) and 3‐hydroxybutyrate (3‐HB) serve as an important alternative energy source for extrahepatic tissues such as the brain, muscle, and heart.^1^, ^2^

Multiple acyl‐CoA dehydrogenase deficiency (MADD; or glutaric aciduria type II; OMIM #231680) is an ultra‐rare (ie, <1:50000) disorder of mitochondrial FAO and amino acid metabolism.^3^ Treatment options include dietary fat and protein restrictions, fasting avoidance, and supplementation with riboflavin, glycine, coenzyme Q10 and L‐carnitine. Although this treatment is generally sufficient in milder MADD, severely affected patients often develop life‐threatening symptoms as cardiomyopathy, leukodystrophy and myopathy.^3^ Since patients with mitochondrial FAO disorders may exhibit impaired ketogenesis, KB supplementation potentially provides a therapeutic option to ensure an adequate supply.^1^, ^4^ Racemic D,L‐3‐HB salt supplementation (ie, ratio D‐3‐HB:L‐3‐HB is 1:1) has been shown to result in clinical improvement of cardiomyopathy, leukodystrophy and hypotonia in severe MADD.^5^, ^6^ The enterally administered quantities ranged between 100 and 2600 mg/kg D,L‐3‐HB salt in one to six daily doses. D,L‐3‐HB can be prescribed as food supplement or as magistral formula prepared by pharmacists, and is currently available as sodium salt, sodium/calcium salt, or mixed salt formulation.^5^, ^6^ Recently, continuous administration of D,L‐3‐hydroxybutyric acid has been advocated as well.^7^

3‐hydroxybutyrate is a chiral molecule and its D‐ and L‐enantiomer can have different pharmacokinetic (PK) and pharmacodynamic (PD) properties.^8^ To date, the lack of a clear clinical and biochemical dose‐efficacy relationship,^6^ and the absence of PK and PD insights of the individual enantiomers, hamper optimal treatment. To bridge this gap, we analyzed circulating D‐3‐HB and L‐3‐HB concentrations in three MADD patients, and characterized the enantiomer‐specific PK in terms of absorption and distribution in healthy rats, following single dose administration of racemic D,L‐3‐HB.

## 2 MATERIALS AND METHODS

### 2.1 Exploratory measurements in three patients with multiple acyl‐CoA dehydrogenase deficiency

We identified three MADD patients who received enteral D,L‐3‐HB for cardiomyopathy, leukodystrophy and myopathy, in addition to their standard (dietary) treatments. The patient characteristics and D,L‐3‐HB treatment details are described in Data S1. Blood samples were obtained at several time points post‐administration and analyzed for D‐3‐HB and L‐3‐HB concentrations. The results of longitudinal urinary metabolite monitoring in patient 1 were analyzed retrospectively.

The exploratory patient studies were performed according to the principles of the Helsinki Declaration, as revised latest in 2013. For patient 1, the Medical Ethical Committee of the University Medical Center Groningen confirmed that the Medical Research Involving Human Subjects Act did not apply for the retrospective data analysis and that official approval of this study by the Medical Ethical Committee was not required (METc 2019/119). The measurements were conducted with parental informed consent, within the context of standard patient care and following the codes of conduct of the FEDERA (Federation of Medical Scientific Institutions). For patient 2, the measurements and clinical review were performed after obtaining informed consent on an IRB‐approved research protocol (COMIRB# 16‐0146). In patient 3, the studies were done within the context of standard patient care.

### 2.2 Animal study

The animal procedures were performed in compliance with EU legislation (Directive 2010/63/EU) and the Public Health Service Policy on Humane Care and Use of Laboratory Animals. The study protocol was approved by the Dutch Central Committee Animal Testing (#AVD1050020172265). All institutional and national guidelines for the care and use of laboratory animals were followed.

Sodium‐D,L‐3‐HB (≥99.0%) was purchased from Sigma‐Aldrich and prepared as solutions in demineralized water (range: 260‐1030 mg sodium‐D,L‐3‐HB/mL ([C_4_H_7_O_3_Na (aq)] = 2.1‐8.2 M)). The concentrations ensured dosing with weight‐based volumes (range: 2.6‐3.4 mL) between 70% and 80% of the recommended maximum volumes for administration via oral gavage.

Twenty‐five healthy, male Wistar rats (Envigo, the Netherlands) were group housed on a reversed 12 hours light – 12 hours dark cycle (lights on at 20.00 pm). Drinking water and standard chow (RM1 diet, Special Diets Services) were provided ad libitum. Experimental studies were performed after 2 weeks of acclimatization. The experimental timing was accommodated to the rat's active period, with the procedures operated under infrared light, starting 1 hour after onset of the dark period.

### 2.3 Experimental design of the animal study

A flowchart and timeline diagram of the experimental design is presented in Figure S1. The animals (mean weight: 414 g, SD: 30 g) were randomly assigned to three experimental groups and one control group. The rats were housed individually. There were no food or drinking water restrictions in order to mimic the patient setting. The animals in the three experimental groups received a single, oral dose of sodium‐D,L‐3‐HB via oral gavage. To allow correct data interpretation, the doses are described in salt‐free amount (ie, approximately 82% of the sodium‐D,L‐3‐HB dose). The low (n = 6), medium (n = 7), and high dose (n = 6) groups received 1579 mg/kg, 3159 mg/kg, and 6317 mg/kg, respectively (ie, 1926, 3852, and 7704 mg/kg of sodium‐D,L‐3‐HB). These doses correspond to the most commonly prescribed doses in humans of 369 mg/kg, 738 mg/kg, and 1476 mg/kg (ie, 450 mg/kg, 900 mg/kg, and 1800 mg/kg of sodium‐D,L‐3‐HB), based on endogenous D‐3‐HB production rates, as described in Data S2.^9^, ^10^ The control group (n = 6) received demineralized water in equivalent volumes, except for two animals in which oral gavage was not possible due to stress.

Before and post‐administration (ie, 20, 40, 60, 90, 120, 150, 180, 240, and 360 minutes), venous blood samples (about 200 μL) were collected into EDTA cups via tail vein nick bleeds. The rats were euthanized by decapitation after deep anesthesia with inhalation isoflurane. The mean time from experiment initiation to termination was 6 hours and 47 minutes (SD: 23 minutes). Brain, heart, liver, and muscle tissue were collected in 10 to 15 minutes.

### 2.4 Enantiomer‐specific analysis of 3‐hydroxybutyrate and 3‐hydroxybutyrate‐carnitine

A detailed description of the sample preparation procedures is presented in Data S3. The concentrations of D‐3‐HB and L‐3HB were determined in processed blood and tissue samples using reversed phase ultra‐performance liquid chromatography‐tandem mass spectrometry (UPLC‐MS/MS), as previously described for the analysis of D‐lactate and L‐lactate.^11^ For patients 2 and 3, the analysis was performed in deproteinized plasma samples. In short, D‐3‐HB and L‐3‐HB were quantified using an isotopically‐labeled internal standard (final concentration 25 μmol/L 3,4,4,4‐^2^H_4_‐3‐HB). After derivatization with diacetyl‐L‐tartaric‐anhydride, the samples were separated on a BEH‐C_18_‐reversed phase column and detected on a Xevo TQ‐S micro (Waters). The conditions of MS/MS are specified in Data S4. The 3‐HB enantiomer concentrations in blood and tissues were expressed in millimole per liter and micromole per gram of protein, respectively. For patient 2, plasma concentrations of D‐3‐HB and AcAc were also analyzed enzymatically using BDH1 as previously described.^12^, ^13^, ^14^, ^15^ The quantitative oxidation of D‐3‐HB to AcAc in the presence of excess BDH1 and NAD^+^ at pH 8.5, was determined by the increase in absorbance of NADH at 340 nm. Conversely, quantitative reduction of AcAc to D‐3‐HB in the presence of excess BDH1 and NADH at pH 7.0, was determined by the decrease in absorbance of NADH at 340 nm.

To study the involvement of the ketones in mitochondrial metabolismupon D,L‐3‐HB administration, we used high‐performance liquid chromatography MS/MS to analyze the tissue concentrations of D‐3‐HB‐ and L‐3‐HB‐carnitine, as derivatives of enantiomer specific 3‐OH‐butyryl‐CoA.^16^, ^17^ The concentrations are expressed in micromole per gram of protein.

### 2.5 Statistical analysis

Patient data are presented in absolute concentrations. Animal data are presented as mean (SD) or in absolute concentrations. The absorption and elimination phases of D‐3‐HB and L‐3‐HB were depicted in (semi‐log) concentration‐time plots. First, the maximum concentration (*C*_max_) and time to reach *C*_max_ (*t*_max_) were derived directly from the experimental data. The area under the concentration‐time curve (AUC_0‐6_) was calculated based on the linear trapezoidal method. Next, the PK parameters were modeled using MW/Pharm pharmacokinetic software version 3.86 (MwPharm, Zuidhorn, the Netherlands),^18^ as described in Data S5. The enantiomer‐specific population PK models were applied to estimate *C*_max_, *t*_max_ and AUC_0‐6_, using Bayesian fitting to the concentration‐time and weight data. The oral absorption rate constant (*k*_A_), volume of distribution to the central compartment (*V*_1_), elimination half‐life (*t*_1/2_), and metabolic clearance (Cl_m_) were derived from the individual parameters. Model performance was evaluated on basis of the Akaike Information Criterion (AIC), the root of the Weighed Sum of Squares (ΣWSS) and visual inspection of the overall goodness‐of‐fit. An adequate model performance allowed estimation of the distribution and elimination parameters.

Inferential statistical analysis of the absorption data from the animal experiment was performed using GraphPad Prism version 7.02 (GraphPad Software, La Jolla, California) and Analyse‐it in Microsoft Excel (Analyse‐it Software, Ltd, Leeds, UK). One‐way analysis of variance test followed by post hoc Bonferroni's multiple comparison test as appropriate, was used to test for differences of the 3‐HB enantiomer parameters between the experimental groups and the control animals. A paired *t* test was used to analyze differences between parameters of both 3‐HB enantiomers per dose group. A *P*‐value of <.05 was considered statistically significant (*P* < .01 after Bonferroni correction for the analyses between both 3‐HB enantiomers per dose group).

## 3 RESULTS

### 3.1 Exploratory measurements in three patients with multiple acyl‐CoA dehydrogenase deficiency

**FIGURE 1:**
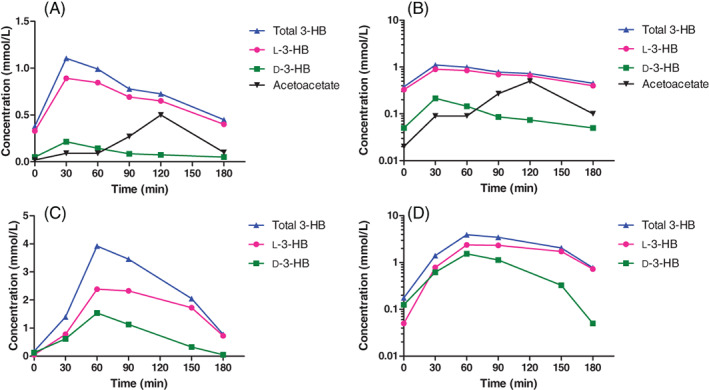
Exploratory measurements in two patients with multiple acyl‐CoA dehydrogenase deficiency after an oral dose of D,L‐3‐hydroxybutyrate. The concentration‐time and semi‐log concentration‐time plots of 3‐HB, D‐3‐HB and L‐3‐HB, and AcAc (if available), determined in plasma after an oral dose of D,L‐3‐HB. For measurements of <0.050, a cutoff value of 0.050 was used. Data of patient 1 is not depicted because the peak concentrations of both enantiomers could not be determined due to measurements at two time points only; she received a salt‐free D,L‐3‐HB dose of 1123 mg/kg/day divided in 5 daily doses; body weight 17.3 kg; 5 years old. Patient 2, A/B, received a multiple salt solution of D,L‐3‐HB at a salt‐free dose of 736 mg/kg/day divided in eight daily doses; body weight 4.5 kg; 3 months old. Patient 3, C/D, received sodium‐D,L‐3‐HB at a salt‐free dose of 820 mg/kg/day of in 4 daily doses; body weight 40.0 kg; 16 years old. Data are presented in absolute concentrations

In all patients, administration of oral D,L‐3‐HB resulted in ketonemia with higher L‐3‐HB‐ compared to D‐3‐HB concentrations, as shown in Table S1. In patient 1, the peak concentrations of both enantiomers could not be determined because of measurements at two time points only. In patients 2 and 3, peak concentrations of both enantiomers were achieved between 30 and 60 minutes after administration and returned toward baseline after 3 hours, as shown in Figure 1. Peak total 3‐HB, D‐3‐HB and L‐3‐HB concentrations ranged between 1.11‐3.92 mmol/L, 0.21‐1.54 mmol/L, and 0.89‐2.38 mmol/L, respectively. In patient 2, following administration of D,L‐3‐HB we also found an increase in AcAc with peak concentrations of 0.40 to 0.50 mmol/L, as shown in Table S1.

Longitudinal urinary metabolite monitoring in patient 1, revealed highly elevated excretions of tricarboxylic acid (TCA) cycle intermediates including citrate, aconitate, alpha‐ketoglutarate, malate, fumarate and succinate, as presented in Figure S2.

### 3.2 Animal study

**FIGURE 2:**
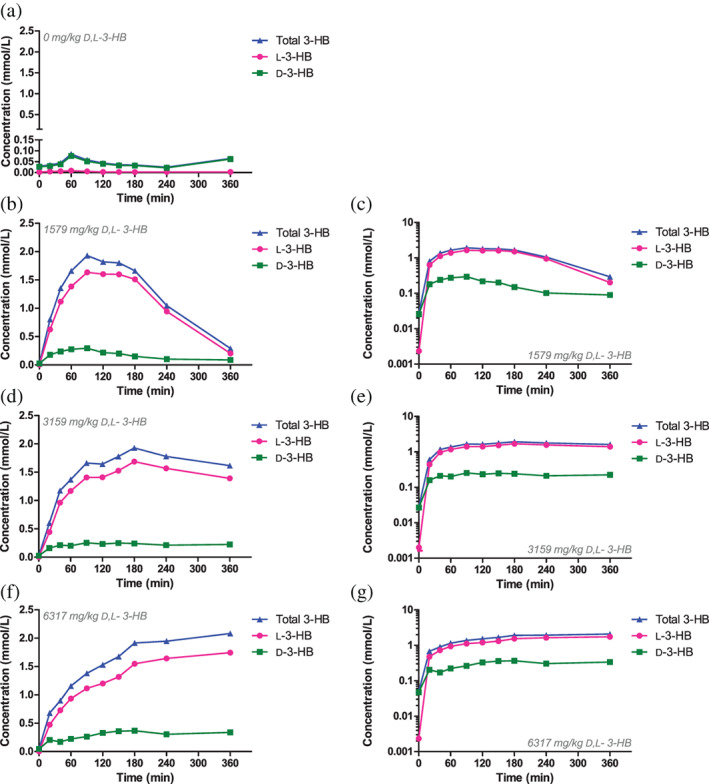
The enantiomer‐specific absorption after a single, oral dose of D,L‐3‐hydroxybutyrate in rats. The blood concentration‐time and semi‐log concentration‐time plots of 3‐HB, D‐3‐HB and L‐3‐HB in the control group (n = 6), A, and after oral sodium‐D,L‐3‐HB at a salt‐free dose of 1579 mg/kg (n = 6), B/C, 3159 mg/kg (n = 7), D/E, and 6317 mg/kg (n = 6), F/G. Data are presented as mean of the respective groups

All animals were included in the analyses. Data from 16 out of 250 samples (control group: n = 12; experimental groups: n = 4) were missing due to inadequate blood collection or sample processing. The D‐3‐HB and L‐3‐HB concentrations in controls were low and stable. Single dose administration of D,L‐3‐HB induced ketonemia in all experimental groups. Maximum total 3‐HB, D‐3‐HB and L‐3‐HB concentrations ranged between 2.1‐2.4 mmol/L, 0.30‐0.50 mmol/L, and 1.88‐1.98 mmol/L, respectively, as shown in Figure 2.

### 3.3 The absorption of D,L‐3‐hydroxybutyrate

The 3‐HB, D‐3‐HB, and L‐3‐HB concentration‐time curves are presented in Figure 2. The PK parameters per administered dose are listed in Table 1. Only for the low‐ and medium dose groups, the absorption and (part of) the elimination phases could be observed, with *C*_max_ reflecting peak concentrations. In all experimental groups, the obtained L‐3‐HB concentrations were substantially higher than D‐3‐HB, with significant differences for *C*_max_ and AUC. The C_max_ and AUC of both D‐3‐HB and L‐3‐HB increased significantly in all experimental groups as compared to the controls. For D‐3‐HB, the trend appeared dose‐dependent (data of controls vs the ascending dose groups for *C*_max_: 0.10 vs 0.30‐0.35‐0.50 mmol/L, and AUC: 14 vs 58‐71‐106 minutes*mmol/L), with significant differences between the *C*_max_ and AUC of the low dose and high dose group, and the medium dose and high dose group. The increase in *C*_max_ and AUC of L‐3‐HB was not dose proportional (data of controls vs ascending dose groups for *C*_max_: 0.01 vs 1.88‐1.92‐1.98 mmol/L, and AUC: 1 vs 380‐454‐479 minutes*mmol/L). There was a significant increment in *t*_max_ of D‐3‐HB and L‐3‐HB upon increased D,L‐3‐HB dosing. The *t*_max_ of D‐3‐HB was lower than that of L‐3‐HB for all administered doses, however, the difference did not reach statistical significance. The elimination of D‐3‐HB and L‐3‐HB demonstrated signs of zero order kinetics (ie, saturable elimination) upon ketonemia, as indicated by the linear parts in the concentration‐time plots and the convex parts in the semi‐log concentration‐time plots in Figures 1 and 2.

**TABLE 1:** Pharmacokinetic parameters of D‐3‐hydroxybutyrate and L‐3‐hydroxybutyrate after a single, oral dose of D,L‐3‐hydroxybutyrate in rats

		D‐3‐HB		L‐3‐HB		Total 3‐HB	
	Parameter	Raw data	PK model	Raw data	PK model	Raw data	PK model
Controls, n = 6 0 mg/kg	*C*_max_	0.10 (0.07)	—	0.01 (0.00)	—	0.10 (0.07)	—
	AUC_0‐6_	14 (3)	—	1 (0)	—	16 (3)	—
Low dose, n = 6 1579 mg/kg	*C*_max_	0.30 (0.03)^a^	0.28 (0.03)	1.88 (0.23)^a^	1.64 (0.24)	2.12 (0.23)	1.96 (0.27)
	*t*_max_	85 (12)	64 (10)	135 (31)	101 (10)	135 (31)	97 (9)
	*t*_1/2_	—	129 (93)	—	71 (6)	—	75 (25)
	AUC_0‐6_	58 (10)^a^	58 (10)	380 (55)^a^	338 (159)	438 (62)	406 (45)
	Cl_m_	—	7.04 (2.25)	—	1.06 (0.11)	—	0.95 (0.14)
	*V*_1_	—	0.42 (0.15)	—	0.04 (0.01)	—	0.04 (0.01)
	*k*_A_	—	2.04 (1.11)	—	0.59 (0.05)	—	0.79 (0.34)
Medium dose, n = 7 3159 mg/kg	*C*_max_	0.35 (0.11)^a^	0.26 (0.07)	1.92 (0.47)^a^	1.61 (0.50)	2.24 (0.50)	1.87 (0.57)
	*t*_max_	167 (104)	106 (70)	227 (101)	233 (95)^b^	223 (104)	175 (87)
	AUC_0‐6_	71 (17)^a^	77 (18)	454 (140)^a^	416 (192)	526 (155)	467 (239)
High dose, n = 6 6317 mg/kg	*C*_max_	0.50 (0.13)^abc^	0.36 (0.10)	1.98 (0.26)^a^	1.71 (0.41)	2.41 (0.37)	2.05 (0.45)
	*t*_max_	245 (94)^b^	211 (120)^b^	340 (49)^bc^	332 (68)^b^	340 (49)	309 (80)
	AUC_0‐6_	106 (29)^abc^	107 (26)^b^	479 (87)^a^	475 (91)	585 (97)	581 (96)

*Note*: Data are presented as mean (SD). For the low and medium dose group, the absorption and (part of) the elimination phases were observed, with *C*_max_ reflecting peak concentrations. Upon high dose D,L‐3‐HB, we only observed the absorption phase, with *C*_max_ possibly not reflecting peak concentrations. Based on the model performance, the distribution and elimination parameters could be estimated for the low dose group only. One‐way analysis of variance test followed by post hoc Bonferroni's multiple comparison test was used to test the 3‐HB enantiomer parameters for significant differences ^a^from control data; ^b^from the low dose group; ^c^from the medium dose group. The results were considered significantly different if *P* < .05. Abbreviations (in alphabetical order): AUC, area under the concentration‐time curve (min*mmol/L); Cl_m_, metabolic clearance (L/h), *C*_max_, maximum concentration (mmol/L); PK, pharmacokinetic; *k*_A_, oral absorption rate constant (h^−1^); *t*_max_, time point at which *C*_max_ is reached (min); *t*_1/2_, elimination half‐life (min); *V*_1_, volume of distribution to the central compartment (L/kg).

**FIGURE 3:**
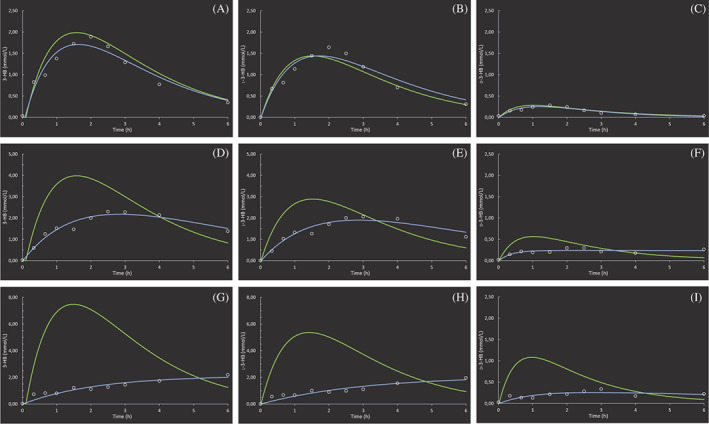
The final, enantiomer‐specific population pharmacokinetic models after a single, oral dose of D,L‐3‐hydroxybutyrate in rats. The modeled blood‐concentration time plots of 3‐HB, D‐3‐HB, and L‐3‐HB after oral sodium D,L‐3‐HB at a salt‐free dose 1579 mg/kg (n = 6), A/B/C, 3159 mg/kg (n = 7), D/E/F, and 6317 mg/kg (n = 6), G/H/I. The green line represents the concentration‐time curve from the population model. The blue line depicts the individual a posteriori Bayesian‐estimated curve for one representative rat of the group, with the dots representing the measured concentrations

The final enantiomer‐specific population PK models and corresponding variables are presented in Figure 3 and Table S2, respectively. The model performance was adequate for the low dose group only, as also demonstrated by the similar curves of the individual‐ and population model data. The modeled PK parameters are presented in Table 1. The *C*_max_ and AUC of L‐3‐HB were significantly higher than those of D‐3‐HB in all experimental groups. For both 3‐HB enantiomers, a dose‐dependent increment in *C*_max_ was not observed (D‐3‐HB: 0.28‐0.26‐0.36; and L‐3‐HB: 1.64‐1.61‐1.71). The trend in AUC appeared dose proportional for D‐3‐HB (58‐77‐107 minutes*mmol/L), with a significant difference between the low and high dose group, whereas for L‐3‐HB this was not observed (338‐416‐475 minutes*mmol/L). Upon increased D,L‐3‐HB dosing, the *t*_max_ of D‐3‐HB and L‐3‐HB increased significantly. The *t*_max_ of D‐3‐HB was lower compared to L‐3‐HB for all dose groups, with the difference reaching statistical significance in the low dose group.

### 3.4 The distribution of D,L‐3‐hydroxybutyrate

**FIGURE 4:**
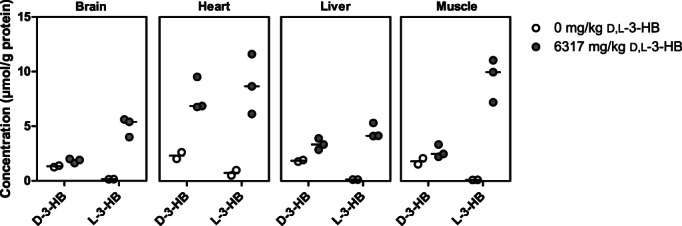
The enantiomer‐specific tissue distribution after a single, oral dose of D,L‐3‐hydroxybutyrate in rats. The concentrations of D‐3‐HB and L‐3‐HB in brain, heart, liver, and muscle of control animals (n = 2) and after oral sodium‐D,L‐3‐HB at a salt‐free dose of 6317 mg/kg (n = 3). Data are presented as scatter dot plots including median values

In control animals (n = 2), all tissue D‐3‐HB concentrations exceeded the L‐3‐HB concentrations (D‐3‐HB vs L‐3‐HB brain: 1.32 (1.26, 1.39) vs 0.15 (0.14, 0.16) μmol/g protein; in heart: 2.33 (2.02, 2.63) vs 0.75 (0.51, 0.99) μmol/g protein; in liver: 1.85 (1.78, 1.92) vs 0.13 (0.13, 0.13) μmol/g protein; and muscle tissue 1.81 (1.53, 2.08) vs 0.10 (0.09, 0.10) μmol/g protein). This reversed upon a single, high dose of D,L‐3‐HB (n = 3) with a considerable increase of L‐3‐HB in all tissues, including notably in brain, whereas the increase in D‐3‐HB was most pronounced in heart and liver tissue, and barely noticeable in brain and muscle, as depicted in Figure 4.

Upon a single, high dose of D,L‐3‐HB, we observed an increase in D‐3‐HB‐ and L‐3‐HB‐carnitine in heart tissue, as demonstrated in Figure S3. The concentrations in brain, liver and muscle tissue were very low, and differences were therefore difficult to interpret. After D,L‐3‐HB administration, there appeared to be an increase of both 3‐HB‐carnitine esters in muscle tissue, and of L‐3‐HB‐carnitine in brain tissue. In liver, we found no changes in D‐3‐HB‐ and L‐3‐HB‐carnitine concentrations upon a single, high dose of D,L‐3‐HB. The percentage increase in D‐3‐HB and L‐3‐HB concentrations was not reflected in the relative changes in D‐3‐HB‐ and L‐3‐HB‐carnitine in the respective tissues.

## 4 DISCUSSION

The field of KB management is rapidly expanding. Knowledge of D,L‐3‐HB PK and PD is essential for its further development, to enable general application, and to improve the chance of beneficial outcomes in patients. Here, we characterized the absorption and distribution of D‐3‐HB and L‐3‐HB following oral administration of racemic D,L‐3‐HB salt. To the best of our knowledge, this is the first UPLC‐MS/MS‐based enantiomer‐specific PK study of D,L‐3‐HB.

Previous preclinical studies generated conflicting results on the capacity of D,L‐3‐HB salts to provoke ketonemia.^19^, ^20^ Here, we demonstrate in MADD patients and in healthy rats, that enteral D,L‐3‐HB salt produces ketonemia with substantially higher concentrations of L‐3‐HB than D‐3‐HB. In rats, the increment in *C*_max_ of L‐3‐HB was non‐dose proportional, whereas a possible dose responsive trend could be noticed for D‐3‐HB. Combined with the prolonged *t*_max_, this could indicate saturated absorption for L‐3‐HB. The heterogeneous patient PK data, with a considerably lower *C*_max_ of D‐3‐HB in the two younger patients, may imply a higher utilization and thus exogenous requirement at a younger age, although individual differences in the uptake capacity, underlying genetic defect and treatment characteristics may also play a role.^10^, ^21^ Timing of the peak 3‐HB enantiomer concentrations in MADD patients appeared optimal between 30 and 60 minutes after administration. The elimination of both 3‐HB enantiomers may be explained by a Michaelis‐Menten process, with saturation upon ketonemia. This is substantiated by the dose‐dependent kinetics in our PK models. Although confirmatory experiments are required, our findings are in line with earlier studies demonstrating a reduction in KB clearance in hyperketotic states, and non‐linear elimination following administration of D‐3‐HB ester.^22^, ^23^ In MADD patients during chronic oral intake, we found that concentrations of D‐3‐HB and L‐3‐HB returned toward baseline concentrations after about 3 hours. Based on our limited patient measurements, we recommend a dosing schedule of six to eight daily administrations upon initiation of D,L‐3‐HB in MADD, which can subsequently be tailored to the individual patient. The place of continuous administration^6^, ^7^ and slow release preparations, for example in the form of a 1,3‐butanediol‐ or glycerol‐derived ester or a polymer,^24^, ^25^, ^26^ remains to be determined.

**FIGURE 5:**
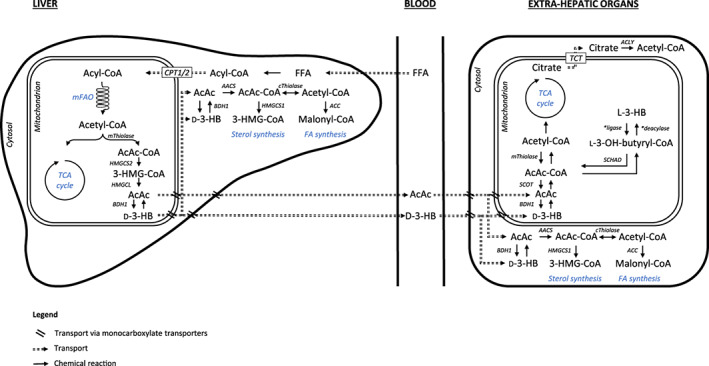
Schematic representation of the proposed production and utilization of ketone bodies. D‐3‐HB is primarily formed in the liver via the 3‐hydroxy‐3‐methylglutaryl‐CoA pathway, whereas the route of endogenous L‐3‐HB synthesis remains to be fully elucidated. In mitochondria, acetoacetyl‐CoA can be converted to L‐3‐HB‐CoA, and subsequently formed to L‐3‐HB via a specific CoA deacylase,^45^ however this conversion does not appear to take place in the liver.^46^ D‐3‐HB is oxidized to AcAc in extrahepatic tissues and subsequently reconverted to two molecules of acetyl‐CoA which enter the Krebs cycle.^35^ In contrast, a specific CoA ligase activates L‐3‐HB to L‐3‐hydroxybutyryl‐CoA after mitochondrial import.^29^ L‐3‐hydroxybutyryl‐CoA is metabolized to acetyl CoA by short‐chain acyl‐CoA dehydrogenase.^33^, ^45^, ^47^ Next to energy generation, non‐oxidative fates of KB may include fatty acid and cholesterol synthesis. Abbreviations (in alphabetical order): 3‐HMG‐CoA, 3‐hydroxy‐3‐methylglutaryl‐CoA; AcAc, acetoacetate; AcAc‐Coa, acetoacetyl‐CoA; AACS, acetoacetyl‐CoA synthetase; ACC, acetyl‐CoA carboxylase; ACLY, adenosine triphosphate citrate lyase; BDH1, D‐3‐hydroxybutyrate dehydrogenase; CoA, coenzyme A; CPT1, carnitine palmitoyltransferase 1; CPT2, carnitine palmitoyltransferase 2; cThiolase, cytosolic thiolase; D‐3‐HB, D‐3‐hydroxybutyrate; FA, fatty acids; FFA, free fatty acids; HMGCL, 3‐hydroxy‐3‐methylglutaryl‐CoA lyase; HMGCS1, 3‐hydroxy‐3‐methylglutaryl‐CoA synthase 1; HMGCS2, 3‐hydroxy‐3‐methylglutaryl‐CoA synthase 2; L‐3‐HB, L‐3‐hydroxybutyrate; L‐3‐OH‐butyryl‐CoA, L‐3‐hydroxybutyryl‐CoA; mFAO, mitochondrial fatty acid oxidation; mThiolase, mitochondrial thiolase; SCHAD, short‐chain 3‐hydroxyacyl‐CoA dehydrogenase; SCOT, succinyl‐CoA‐3‐oxoacid‐CoA transferase; TCA, tricarboxylic acid; TCT, tricarboxylate transport

We observed low L‐3‐HB concentrations in blood and tissues of control animals compared to D‐3‐HB, with the highest content in the heart (ie, L‐3‐HB made up about 10%, 10%, 24%, 6%, and 5% of the total 3‐HB concentration in blood, brain, heart, liver and muscle, respectively). Whether this represents normal metabolism or is caused by hydrolysis of L‐3‐OH‐butyryl‐CoA originating from FAO cannot be determined based on our data. However, our results are in line with previous studies.^27^, ^28^ Upon high dose D,L‐3‐HB, the most profound rise in D‐3‐HB was observed in heart and liver. The lower concentrations in brain and muscle may indicate an increased utilization of D‐3‐HB or a limited uptake. L‐3‐HB concentrations increased extensively in all tissues, with the highest concentrations in heart and muscle. Although the organ‐specific effects of D‐3‐HB and L‐3‐HB remain to be elucidated, the distinct PK profiles suggest that D‐3‐HB and L‐3‐HB are differentially metabolized. Figure 5 depicts a schematic representation of the proposed utilization routes. It can be hypothesized that upon oral D,L‐3‐HB administration, D‐3‐HB and L‐3‐HB are absorbed from the gut to the portal system. Transport into cells and mitochondria can occur via monocarboxylate transporters (MCT).^29^ The higher increment in L‐3‐HB may be explained by a slower tissue import or metabolization, as has been demonstrated by labeled ^14^CO_2_ expiration in rats.^30^ As for the tissue import, enantiomer selectivity of MCT may also play a role.^31^ Finally, the potential impact of hepatic first‐pass metabolism on both 3‐HB enantiomers remains unknown.

In mitochondrial FAO disorders, insufficient availability of *endogenous* KB renders patients vulnerable to energy deficiency and the accumulation of toxic metabolites, and furthermore impairs cholesterol synthesis, which is essential for myelin formation.^32^ Administration of *exogenous* D,L‐3‐HB potentially bypasses the disturbed ketogenesis under these conditions. It can be hypothesized that in peripheral tissues, D‐3‐HB oxidation is mainly responsible for the beneficial actions of D,L‐3‐HB supplementation. L‐3‐HB may predominantly act as a substrate for sterol and fatty acid synthesis in the central nervous system, where the key enzymes responsible for its metabolism are highly expressed.^30^, ^33^ The presence of an increased concentration of L‐3‐HB in brain after exogenous administration indicates that this metabolite can cross the blood brain barrier and reach brain tissue, which would be necessary for a white matter therapeutic. L‐3‐HB‐carnitine appeared to increase in heart, brain, and muscle tissue upon D,L‐3‐HB administration, indicating the use of L‐3‐HB in metabolism of these organs. Besides its roles in intermediate metabolism, endogenous 3‐HB exhibits several intracellular signaling functions of which enantiomer‐specificity is not fully known.^34^, ^35^ In addition to posttranslational histone modification that can modulate gene expression levels, endogenous 3‐HB activates the hydroxycarboxylic acid receptor 2, which reduces lipolysis and has anti‐inflammatory and neuroprotective effects.^34^, ^35^ It also inhibits the neuronal vesicular glutamate transporter 2, thereby potentially reducing excitatory glutamate neurotransmission and the generation of reactive oxygen species.^34^, ^35^, ^36^ The possible downstream effects of D,L‐3‐HB on intercellular signaling remain to be systematically investigated. Currently, MADD patients are generally prescribed racemic D,L‐3‐HB salts. The most effective D‐3‐HB:L‐3‐HB ratio may depend on (organ‐specific) treatment indications. It can be hypothesized that an excess of D‐3‐HB might apply for fuel‐derived symptoms, such as cardiomyopathy, whereas increasing L‐3‐HB may be indicated for leukodystrophy given the evidence for brain metabolism. At present, the correlation between blood 3‐HB enantiomer concentrations and organ‐specific clinically meaningful outcomes remains to be established.

In patient 2, we demonstrated increased AcAc concentrations with a D‐3‐HB:AcAc ratio of below one, while it usually approximates 2:1 to 3:1.^21^, ^37^ We also found a profound rise in urinary TCA cycle intermediates in patient 1 upon higher dosing and long‐term treatment with D,L‐3‐HB. These exploratory findings may indicate the oxidation of *exogenous* D,L‐3‐HB and its contribution to the TCA cycle, causing an increased flux. Other explanations for the increased excretion of TCA cycle intermediates include an inhibited renal reabsorption of carboxylic acids, or a disturbed entry of TCA intermediates into the mitochondria. Paradoxically, the risk of a dysfunctional TCA cycle upon excessive KB oxidation should also be considered. This could potentially be caused by (a) sequestration of CoA, (b) NAD^+^ depletion, or (c) because KB oxidation is essentially a cataplerotic process without replenishment of TCA cycle intermediates. However, first, sequestration of CoA would lead to inhibition at the level of alpha‐ketoglutarate dehydrogenase, and is therefore not expected to result in an overall increased excretion of TCA intermediates, including those after alpha‐ketoglutarate. Second, D‐3‐HB metabolism toward acetyl‐CoA has a 75% lower NAD^+^ consumption compared to glucose, rendering depletion less likely.^38^ In fact, an increased NAD^+^:NADH ratio has been proposed as a key mechanism for the beneficial effects of KB therapies.^39^ Third, cataplerotic KB oxidation can be compensated by an adequate supply of anaplerotic carbohydrates and amino acids to maintain the TCA cycle flux.^40^, ^41^, ^42^, ^43^, ^44^ Finally, D,L‐3‐HB supplementation has been clinically beneficial in MADD patients.^6^ The potential acute harm of a dysfunctional TCA cycle therefore does not seem applicable. Nonetheless, the significance of these exploratory findings should be investigated in future studies, possibly combined with computational modeling.

Several study limitations deserve discussion. The patient measurements were exploratory. They reflect the 3‐HB levels during chronic oral administration as would be typically found during its clinical use. They do not provide for the derivation of pharmacokinetic parameters after single oral dosing. Possible confounding factors, for example by the standard (dietary) treatments, have not been accounted for. In the preclinical study, first, oral gavage was not performed in two controls due to stress, and there was a low power for the tissue analyses. Second, to reflect the target population, the experiments were performed in the non‐fasted state. Although the endogenous KB production is expected to be low, it may have confounded our results.^2^, ^35^ Third, potential interconversion between D‐3‐HB, L‐3‐HB and AcAc has not been accounted for. Use of separate ^13^C‐labeled D‐3‐HB and L‐3‐HB would allow to study the enantiomer‐specific 3‐HB metabolism in depth. Fourth, for further validation of the PK models, it would be helpful to obtain data for >360 minutes after D,L‐3‐HB administration and upon intravenous dosing to calculate the absorption rate. Finally, differences regarding the diseased vs healthy state, and chronic administration vs single dosing, prevented the comparison of the identified PK profiles in patients and rats. Execution of these studies in a preclinical model for MADD is key to investigate the consequences of endogenous KB shortage on the PK of D,L‐3‐HB, and to further establish the effects on clinical outcome.

In conclusion, our study provides a proof of principle on the absorption and distribution of D‐3‐HB and L‐3‐HB upon a single, oral dose of racemic D,L‐3‐HB. These findings will contribute to further improvement of D,L‐3‐HB treatment in patients with inborn errors of metabolism in which ketogenesis is disturbed, such as MADD.

## CONFLICT OF INTEREST

The authors declare that they have no potential conflicts of interest.

## AUTHOR CONTRIBUTIONS

Conceptualization: Willemijn J. van Rijt, Johan L. K. Van Hove, Maaike H. Oosterveer, Terry G. J. Derks; Methodology: Willemijn J. van Rijt, Johan L. K. Van Hove, Rick Havinga, Derk P. Allersma, Tanja R. Zijp, Maaike H. Oosterveer, Terry G. J. Derks; Software: Derk P. Allersma, Tanja R. Zijp; Validation: Frédéric M. Vaz, Derk P. Allersma, Tanja R. Zijp; Investigation: Willemijn J. van Rijt, Frédéric M. Vaz, Rick Havinga, Jirair K. Bedoyan, M. R. Heiner‐Fokkema; Formal analysis: Willemijn J. van Rijt, Johan L. K. Van Hove, Derk P. Allersma, Tanja R. Zijp, Maaike H. Oosterveer, Terry G. J. Derks; Data interpretation: Willemijn J. van Rijt, Johan L. K. Van Hove, Frédéric M. Vaz, Derk P. Allersma, Tanja R. Zijp, Rebecca Heiner‐Fokkema, Dirk‐Jan Reijngoud, Michael T. Geraghty, Ronald J. A. Wanders, Maaike H. Oosterveer, Terry G. J. Derks; Resources: Johan L. K. Van Hove, Frédéric M. Vaz, Rick Havinga, Derk P. Allersma, Jirair K. Bedoyan, Rebecca Heiner‐Fokkema, Dirk‐Jan Reijngoud, Michael T. Geraghty, Ronald J. A. Wanders, Maaike H. Oosterveer, Terry G. J. Derks; Data curation: Willemijn J. van Rijt, Johan L. K. Van Hove, Derk P. Allersma, Tanja R. Zijp, Maaike H. Oosterveer, Terry G. J. Derks; Writing—Original Draft: Willemijn J. van Rijt, Johan L. K. Van Hove, Maaike H. Oosterveer, Terry G. J. Derks; Writing—Review and Editing: Willemijn J. van Rijt, Johan L. K. Van Hove, Frédéric M. Vaz, Rick Havinga, Derk P. Allersma, Tanja R. Zijp, Jirair K. Bedoyan, Rebecca Heiner‐Fokkema, Dirk‐Jan Reijngoud, Michael T. Geraghty, Ronald J. A. Wanders, Maaike H. Oosterveer, Terry G. J. Derks; Visualization: Willemijn J. van Rijt, Johan L. K. Van Hove, Derk P. Allersma, Tanja R. Zijp, Rebecca Heiner‐Fokkema; Supervision: Johan L. K. Van Hove, Maaike H. Oosterveer, Terry G. J. Derks; Project administration: Willemijn J. van Rijt, Maaike H. Oosterveer, Terry G. J. Derks; Funding acquisition: Willemijn J. van Rijt, Maaike H. Oosterveer, Terry G. J. Derks. All authors read and approved the final manuscript. Willemijn J. van Rijt, Johan L. K. Van Hove, Maaike H. Oosterveer, and Terry G. J. Derks accept full responsibility for the work and conduct of the study, had access to the data, and controlled the decision to publish.

## ETHICS APPROVAL

The exploratory patient studies were performed according to the principles of the Helsinki Declaration, as revised latest in 2013. For patient 1, the Medical Ethical Committee of the University Medical Center Groningen confirmed that the Medical Research Involving Human Subjects Act did not apply for the retrospective data analysis and that official approval of this study by the Medical Ethical Committee was not required (METc 2019/119). The measurements were conducted with parental informed consent, within the context of standard patient care and following the codes of conduct of the FEDERA (Federation of Medical Scientific Institutions). For patient 2, the measurements and clinical review were performed after obtaining informed consent on an IRB‐approved research protocol (COMIRB# 16‐0146). In patient 3, the studies were done within the context of standard patient care.

The animal procedures were performed in compliance with EU legislation (Directive 2010/63/EU) and the Public Health Service Policy on Humane Care and Use of Laboratory Animals. The study protocol was approved by the Dutch Central Committee Animal Testing (#AVD1050020172265). All institutional and national guidelines for the care and use of laboratory animals were followed.

## Supporting information

**Supplementary Data S1** Summarized patient details and treatment characteristics.
**Supplementary Data S2**. The conversion between human and animal doses of D,L‐3‐hydroxybutyrate based on endogenous ketone body production rates.
**Supplementary Data S3**. The procedures for sample preparation.
**Supplementary Data S5**. Specification of the population pharmacokinetic model development.
**Supplementary Table S1**. Observations in three patients with multiple acyl‐CoA dehydrogenase deficiency after an oral dose D,L‐3‐hydroxybutyrate.
**Supplementary Table S2**. Variables of the final enantiomer‐specific population pharmacokinetic models after a single, oral dose of D,L‐3‐hydroxybutyrate in rats.
**Supplementary Figure S1**. Flowchart and timeline diagram of the experimental design of the animal study.
**Supplementary Figure S2**. Urinary excretion of tricarboxylic acid cycle intermediates upon treatment with D,L‐3‐hydroxybutyrate in a patient with multiple acyl‐CoA dehydrogenase deficiency.
**Supplementary Figure S3**. The enantiomeric‐specific tissue distribution of 3‐hydroxybutyrylcarnitine after a single, oral dose of D,L‐3‐hydroxybutyrate in rats.

## Data Availability

Access to the presented data is available upon request.
