# Supplementary material for: Enantiomer‐specific pharmacokinetics of D,L‐3‐hydroxybutyrate: Implications for the treatment of multiple acyl‐CoA dehydrogenase deficiency
Source: J Inherit Metab Dis. 2021 Feb 15;44(4):926–38. doi: 10.1002/jimd.12365 (PMC8359440; doi:10.1002/jimd.12365)
Supplement: Supplementary file 1 — Supplementary Data S1 Summarized patient details and treatment characteristics. Supplementary Data S2. The conversion between human and animal doses of D,L‐3‐hydroxybutyrate based on endogenous ketone body production rates. Supplementary Data S3. The procedures for sample preparation. Supplementary Data S5. Specification of the population pharmacokinetic model development. Supplementary Table S1. Observations in three patients with multiple acyl‐CoA dehydrogenase deficiency after an oral dose D,L‐3‐hydroxybutyrate. Supplementary Table S2. Variables of the final enantiomer‐specific population pharmacokinetic models after a single, oral dose of D,L‐3‐hydroxybutyrate in rats. Supplementary Figure S1. Flowchart and timeline diagram of the experimental design of the animal study. Supplementary Figure S2. Urinary excretion of tricarboxylic acid cycle intermediates upon treatment with D,L‐3‐hydroxybutyrate in a patient with multiple acyl‐CoA dehydrogenase deficiency. Supplementary Figure S3. The enantiomeric‐specific tissue distribution of 3‐hydroxybutyrylcarnitine after a single, oral dose of D,L‐3‐hydroxybutyrate in rats. [file JIMD-44-926-s001.docx]

**Supplementary material**

**Supplementary Data 1. Summarized patient details and treatment characteristics.**

We identified three MADD-patients that received enteral D,L-3-HB (i.e. oral or gastric) based on the best published clinical evidence.^1-3^

Indications for D,L-3-HB treatment in patient 1, with neonatal onset MADD, included cardiomyopathy, leukodystrophy and myopathy. She suffered from homozygous mutations in *ETFA* (c.1-40G>A). From the age of seven months, racemic sodium-D,L-3-HB (Sigma-Aldrich) was introduced at a salt-free D,L-3-HB dose of 369 mg/kg/day (i.e. approximately 82% of the sodium-D,L-3-HB dose of 450 mg/kg/day) which was increased to 738 mg/kg/day (sodium-D,L-3-HB dose of 900 mg/kg/day) after one week. The maximum salt-free dose during the course of treatment was 2132 mg/kg/day (sodium-D,L-3-HB dose of 2600 mg/kg/day). This dose was gradually weaned to 1123 mg/kg/day (sodium-D,L-3-HB dose of 1370 mg/kg/day), divided in five daily doses at the time of blood sampling.

In patient 2, D,L-3-HB treatment was started on the eight day of life for cardiomyopathy and leukodystrophy. Molecular analysis identified compound heterozygous mutations in *ETFDH* (c.121C>T and c.1117T>C). The infant received a mixed salt solution (sodium, potassium, calcium and magnesium) of racemic D,L-3-HB (Trumacro^TM^ Nutrition), initially at a salt-free D,L-3-HB dose of 736 mg/kg/day (i.e. approximately 66% of the D,L-3-HB salt solution dose of 1115 mg/kg/day) provided in doses every three hours, and later 896 mg/kg/day (D,L-3-HB salt solution dose of 1357 mg/kg/day).

Patient 3 suffers from later onset MADD with compound heterozygous mutations in *ETFDH* (c.51dupT and c.940G>A).^3^ From the age of four years and three months, racemic sodium-D,L-3-HB (Veriton Pharma, Fischer Scientific) was introduced at a salt-free D,L-3-HB dose 410 mg/kg/day (sodium-D,L-3-HB dose of 500 mg/kg/day) because of leukodystrophy with subsequent regression of the lesions. At the age of 18 years, the treatment was dosed at 820 mg/kg/day (sodium-D,L-3-HB dose of 1000 mg/kg/day) divided in four daily doses.

**Supplementary Data 2. The conversion between human and animal doses of D,L-3-hydroxybutyrate based on endogenous ketone body production rates.**

To allow correct data interpretation, the doses are described in salt-free amount (i.e. approximately 82% of the sodium-D,L-3-HB dose). Animal doses were based on the most commonly prescribed D,L-3-HB doses in MADD-patients of 369 mg/kg, 738 mg/kg, and 1476 mg/kg (i.e. 450 mg/kg, 900 mg/kg, and 1800 mg/kg sodium-D,L-3-HB). Human doses were converted to rat doses on the basis of endogenous ketone body production rates as determined by D-(-)-3-hydroxy[4,4,4-2H3]butyrate tracer studies.^4,5^ In neonates and infants, the endogenous ketone body production approximates 18.6 μmol/kg/min upon 3.5 to 8.5 hours of fasting.^5^ In overnight-fasted Wistar rats, endogenous KB production rate is circa 17.9 μmol/~225g/min, i.e. 79.6 μmol/kg/min.^4^ Based on these turnover rates, a conversion factor of *4.28 was calculated. This resulted in the corresponding animal doses of salt-free D,L-3-HB of 1579 mg/kg (low dose; i.e. sodium-D,L-3-HB dose of 1926 mg/kg), 3159 mg/kg (medium dose; i.e. sodium-D,L-3-HB dose of 3852 mg/kg), and 6317 mg/kg (high dose; i.e. sodium-D,L-3-HB dose of 7704 mg/kg).

Based on allometric conversion between animal and human doses, the used animal doses would be equivalent to circa 653 mg/kg, 1307 mg/kg, and 2614 mg/kg salt-free D,L-3-HB for the low, medium and high dose respectively, for an infant weighing 6 kg.^6^

**Supplementary Data 3. The procedures for sample preparation.**

*Blood samples*

Immediately after collection, 100 μL whole-blood from the EDTA cups was deproteinized with an equal volume of 1.0 M perchloric acid. The solution was gently mixed by finger tapping, stored on ice and centrifuged at 1700 xg for 10 minutes at 4 ˚C. The supernatant was centrifuged again under the same conditions, pipetted off and stored at -80 ˚C for 3-HB enantiomer analysis. For patients 2 and 3, plasma was obtained from EDTA and lithium-heparin cups, respectively, and stored at -80 ^°^C during shipment and until further processing for the 3-HB enantiomer analysis. The samples for the analysis of AcAc in patient 2 were immediately deproteinized upon arrival in the laboratory, stored at -70 ˚C until shipment to the analyzing laboratory on dry ice, where they were also stored at -70 ˚C until analysis which occurred within one week.

*Tissue samples*

Collected tissue samples were rapidly freeze-clamped in liquid nitrogen and stored at -80 ˚C. Frozen tissue samples were crushed in liquid nitrogen. Homogenates were prepared by adding phosphate-buffered saline solution (pH 7.4) to frozen powder aliquots in 20% weight:volume ratio. The homogenates were sonified at 11-12 W for 30 seconds (pulse time of 1 second), with the tube immersed in ice. Subsequently, the supernatants were collected after 10 minutes of centrifugation at 12800 rpm at 4 ˚C. Protein concentrations were analyzed using a Pierce BCA protein assay kit. From the remaining sample, 100 μL was deproteinized using an equal volume of 1.0 M perchloric acid. The subsequent work-up was identical as described for the blood samples. Supernatants were stored at -80 ˚C for 3-HB enantiomer analysis.

For the analysis of the concentrations of D-3-HB- and L-3-HB-carnitine in the collected tissue samples, homogenates were prepared by adding 0.9% NaCl to powder aliquots which were subsequently sonified with a tip sonicator. The protein concentration of the homogenate was measured with a Pierce BCA protein assay kit and analyzed essentially as described previously,^7^ and the D-3-HB and L-3-HB-carnitine levels were calculated using a C4-carnitine internal standard and expressed micromole per gram of protein.

**Supplementary Data 4. Specification of the conditions of mass spectrometry.**

Column: Waters BEH-C_18_-column (100 x 2.1mm, 1.7 µM particles)

Eluent A: 1.5 mM NH4-formate, pH 3.6

Eluent B: acetonitril

Gradient:

- Initial flow 0.5 ml/min, 99.9% A
- 3.00 min 0.5 ml/min, 94% A
- 4.00 min 0.5 ml/min, 60 % A
- 4.10 min 0.5 ml/min, 99.9% A
- 6.00 min 0.5 ml/min, 99.9% A

Run time: 6 min

Injection volume: 10 µL

MS/MS:

- capillary voltage: 1.5 kV
- Source temperature: 150 °C
- Desolvation temperature: 450 °C
- Cone gas flow: 50 L/hour
- Desolvation gas flow: 900 L/hour

MRM of DATAN derivates (diacetyl-L-tartaric anhydride):

- 3-hydroxybutyric acid: 319 > 103, cone voltage: 15 V, collision energy: 10 eV
- 3-hydroxybutyric acid-d4: 323 > 107, cone voltage: 15 V, collision energy: 10 eV

**Supplementary Data 5. Specification of the population pharmacokinetic model development.**

Modeling was performed using MW/Pharm pharmacokinetic software version 3.86 (MwPharm, Zuidhorn, the Netherlands).^8^ The initial models were derived by automated linear curve stripping of data from a rat in the lowest dose group with the KinStrip module. All curves could subsequently be adequately fitted in a non-linear one compartment model in the KinFit module, with estimates for the linear metabolic clearance (Cl_m_), oral absorption rate constant (*k_A_*), volume of distribution of central compartment (V_1_), and absorption lag-time (t_lag_; for total 3-HB only). Since there was no data available upon intravenous D,L-3-HB administration, the bioavailability (F) was fixed at 1. Essay error was set at the level of 0.05. The initial models were further developed into population models by non-Bayesian fitting, followed by Iterative Two-Stage Bayesian analysis. The concentration-time, dosing, and weight data were used from the low dose group only, as the medium and high dose group did not have sufficient data on both the absorption and elimination phases.

The model performance was evaluated in each developmental step on the basis of the Akaike Information Criterion (AIC), weighted sum of squares of the residuals divided by the degrees of freedom (ƩWSS/df) and visual inspection of the overall goodness-of-fit. The model performance was assessed as adequate for the low dose group only. The models were therefore unfit to estimate the distribution and elimination parameters for the medium and high dose groups.

**Supplementary Table 1. Observations in three patients with multiple acyl-CoA dehydrogenase deficiency after an oral dose D,L-3-hydroxybutyrate.**

|  | **Salt-free D,L-3-HB dose (mg/kg/day)** | **Time**  **(min)** | **D-3-HB**  **(mmol/L)** | **L-3-HB**  **(mmol/L)** | **Total 3-HB**  **(mmol/L)** | **AcAc (mmol/L)** |
| --- | --- | --- | --- | --- | --- | --- |
| **Patient 1** | 1123 in 5 daily doses | -20 | 0.129 | 0.492 | 0.621 | ND |
|  |  | 60 | 0.187 | 0.521 | 0.708 |  |
| **Patient 2** | 736 in 8 daily doses | 0 | 0.050^a^ | 0.329 | 0.379 | 0.02 |
|  |  | 30 | 0.214 | 0.892 | 1.106 | 0.09 |
|  |  | 60 | 0.145 | 0.846 | 0.991 | 0.09 |
|  |  | 90 | 0.086 | 0.692 | 0.778 | 0.27 |
|  |  | 120 | 0.074 | 0.651 | 0.725 | 0.50 |
|  |  | 180 | 0.050^a^ | 0.400 | 0.450 | 0.10 |
|  | 896 in 8 daily doses | 0 | 0.050^a^ | 0.305 | 0.355 | 0.17 |
|  |  | 40 | 0.289 | 1.666 | 1.955 | 0.40 |
|  |  | 80 | 0.050^a^ | 1.074 | 1.124 | 0.37 |
| **Patient 3** | 820 in 4 daily doses | 0 | 0.125 | 0.050^a^ | 0.175 | ND |
|  |  | 30 | 0.617 | 0.783 | 1.400 |  |
|  |  | 60 | 1.538 | 2.384 | 3.922 |  |
|  |  | 90 | 1.130 | 2.326 | 3.456 |  |
|  |  | 150 | 0.327 | 1.725 | 2.052 |  |
|  |  | 180 | 0.050^a^ | 0.726 | 0.776 |  |

The concentrations of 3-HB, D-3-HB and L-3-HB, determined in supernatants of deproteinized blood for patient 1 and in plasma for patient 2 and 3, after an oral dose of D,L-3-HB. Acetoacetate concentrations were only measured in patient 2. ^a^For measurements of <0.050, a cutoff value of 0.050 was used. Patient 1 received a salt-free D,L-3-HB dose of 1123 mg/kg/day (sodium-D,L-3-HB dose of 1370 mg/kg/day; body weight 17.3 kg; 5 years old). Patient 2 received a salt-free D,L-3-HB dose of 736 mg/kg/day (D,L-3-HB salt solution dose of 1115 mg/kg /day; body weight 4.5 kg; 3 months old), and later 896 mg/kg/day (D,L-3-HB salt solution dose 1357 mg/kg/day; body weight 5.2 kg; 4 months old). Patient 3 received 820 mg/kg/day of salt-free D,L-3-HB (sodium-D,L-3-HB dose of 1000 mg/kg/day; body weight 40.0 kg; 16 years old). Abbreviations: ND, not determined.

**Supplementary Table 2. Variables of the final enantiomer-specific population pharmacokinetic models after a single, oral dose of D,L-3-hydroxybutyrate in rats.**

| **Population parameter** | **D-3-HB** | **L-3-HB** | **Total 3-HB** |
| --- | --- | --- | --- |
| **Cl_m_** | 8.27 (3.02) | 1.29 (0.13) | 0.95 (0.13) |
| **V_1_** | 0.39 (0.16) | 0.04 (0.01) | 0.04 (0.01) |
| ***k_A_*** | 1.74 (1.19) | 0.59 (0.05) | 0.79 (0.35) |
| **T_lag_** | - | - | 0.09 (0.08) |
| **F** | 100 (fixed) | 100 (fixed) | 100 (fixed) |

Data are presented as mean (SD). Abbreviations (in alphabetical order): Cl_m_, metabolic clearance (L/h), F, biological availability (%), *k*_A_, absorption constant of oral dose (h^-1^), T_lag_, lagtime (h), V_1_, volume of distribution of central compartment (L/kg).

A)

B)

**
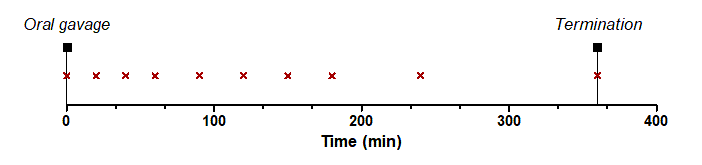
**

**Supplementary Figure 1. Flowchart and timeline diagram of the experimental design of the animal study.**

The animals were randomly assigned to three experimental groups and one control group, as depicted in the flowchart (A). The timelime diagram (B) demonstrates a schematic representation of the experimental design. x = collection of blood sample via tail vein nick bleed. Upon termination, brain, heart, liver, and muscle tissue were collected in 10 to 15 minutes**.**


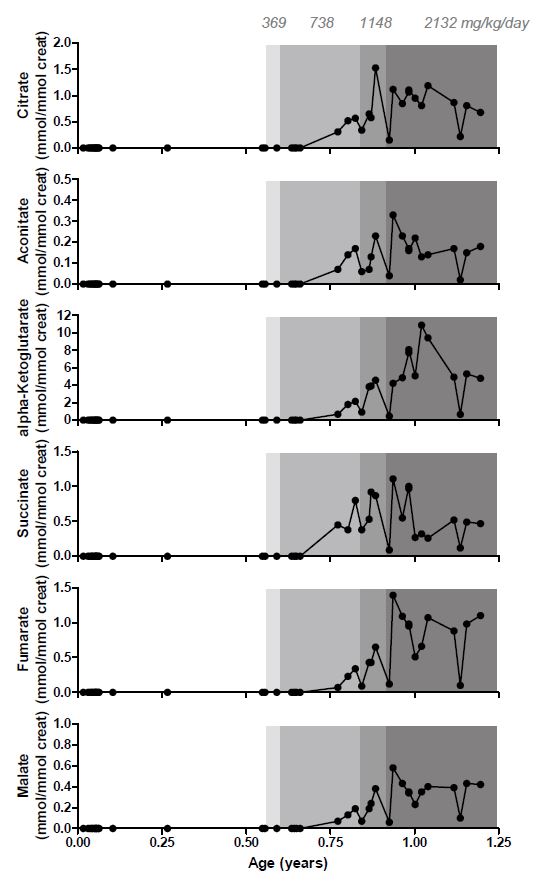


**Supplementary Figure 2. Urinary excretion of tricarboxylic acid cycle intermediates upon treatment with D,L-3-hydroxybutyrate in a patient with multiple acyl-CoA dehydrogenase deficiency.**

Concentrations of tricarboxylic acid cycle intermediates in urine during the course of D,L-3-HB treatment in patient 1. Shaded areas represent the salt-free D,L-3-HB doses between 369 and 2132 mg/kg/day (i.e. sodium-D,L-3-HB dose of 450 to 2600 mg/kg/day).


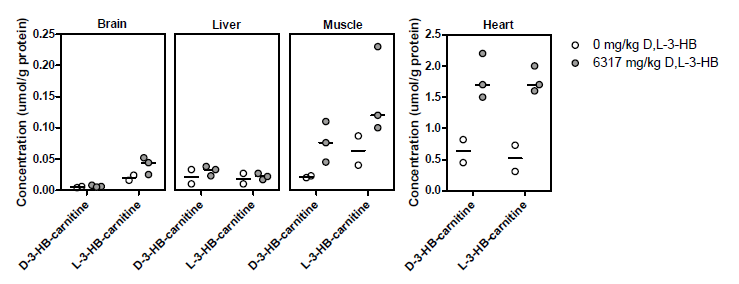


**Supplementary Figure 3. The enantiomeric-specific tissue distribution of 3-hydroxybutyrylcarnitine** **after a single, oral dose of D,L-3-hydroxybutyrate in rats.**

The concentrations of D-3-HB- and L-3-HB-carnitine in brain, liver, muscle and heart of control animals (n = 2) and after oral sodium-D,L-3-HB at a salt-free dose of 6317 mg/kg (n = 3). Data are presented as scatter dot plots including median values.

**References**

1. Van Hove JL, Grunewald S, Jaeken J, et al. D,L-3-hydroxybutyrate treatment of multiple acyl-CoA dehydrogenase deficiency (MADD). *Lancet*. 2003;361(9367):1433-1435.

2. Van Rijt WJ, Heiner-Fokkema MR, du Marchie Sarvaas GJ, et al. Favorable outcome after physiologic dose of sodium-D,L-3-hydroxybutyrate in severe MADD. *Pediatrics*. 2014;134(4):e1224-8.

3. van Rijt WJ, Jager EA, Allersma DP, et al. Efficacy and safety of D,L-3-hydroxybutyrate (D,L-3-HB) treatment in multiple acyl-CoA dehydrogenase deficiency. *Genet Med*. 2020; 22(5):908-916.

4. Bougneres PF, Balasse EO, Ferre P, Bier DM. Determination of ketone body kinetics using a D-(-)-3-hydroxy[4,4,4-2H3]butyrate tracer. *J Lipid Res*. 1986;27(2):215-220.

5. Bougneres PF, Lemmel C, Ferre P, Bier DM. Ketone body transport in the human neonate and infant. *J Clin Invest*. 1986;77(1):42-48.

6. Nair AB, Jacob S. A simple practice guide for dose conversion between animals and human. *J Basic Clin Pharm*. 2016;7(2):27-31.

7. Herzog K, van Lenthe H, Wanders RJA, Vaz FM, Waterham HR, Ferdinandusse S. Identification and diagnostic value of phytanoyl- and pristanoyl-carnitine in plasma from patients with peroxisomal disorders. *Mol Genet Metab*. 2017;121(3):279-282.

8. Proost JH, Meijer DK. MW/pharm, an integrated software package for drug dosage regimen calculation and therapeutic drug monitoring. *Comput Biol Med*. 1992;22(3):155-163.
